# Supplementary material for: Intake of Antioxidant Vitamins and Minerals in Relation to Body Composition, Skin Hydration and Lubrication in Young Women
Source: Antioxidants (Basel). 2021 Jul 12;10(7):1110. doi: 10.3390/antiox10071110 (PMC8301013; doi:10.3390/antiox10071110)
Supplement: Supplementary file 1 [file antioxidants-10-01110-s001.zip › antioxidants-1269588-supplementary.pdf]

antioxidants

MDPI

Supplementary material

Intake of Antioxidant Vitamins and Minerals in Relation to Body Composition, Skin Hydration and Lubrication in Young Women

Anna Puścion-Jakubik *, Renata Markiewicz-Żukowska, Sylwia K. Naliwajko, Krystyna J. Gromkowska-Kępka, Justyna Moskwa, Monika Grabia, Anita Mielech, Joanna Bielecka, Elżbieta Karpińska, Konrad Mielcarek, Patryk Nowakowski and Katarzyna Socha

Department of Bromatology, Faculty of Pharmacy with the Division of Laboratory Medicine, Medical University of Białystok, Mickiewicza 2D Street, 15-222 Białystok, Poland; renmar@poczta.onet.pl (R.M.Ż.), sylwia.naliwajko@umb.edu.pl (S.K.N), krystyna.gromkowska.kepka@gmail.com (K.G.K), justyna.moskwa@umb.edu.pl (J.M.), monika.grabia@umb.edu.pl (M.G.), anita.mielech@umb.edu.pl (A.M.), joanna.bielecka@umb.edu.pl (J.B.), elzbieta.karpinska@umb.edu.pl (E.K.), konrad.mielcarek@umb.edu.pl (K.M.), patryk.nowakowski@umb.edu.pl (P.N.), katarzyna.socha@umb.edu.pl (K.S.)

* Correspondence: anna.puscion-jakubik@umb.edu.pl; Tel.: +48-8574-854-69

Table S1. Correlations between the consumption of ingredients with antioxidant activity ($p < 0.001$).

Factors	A	C	D	E	Cu	Mn	Zn
**A**	-	0.46	0.39	0.56	0.52	0.27	0.46
**C**	0.46	-	-	0.51	0.39	0.32	0.32
**D**	0.39	-	-	0.38	0.37	-	0.41
**E**	0.56	0.51	0.38	-	0.58	0.35	0.52
**Cu**	0.52	0.39	0.37	0.58	-	0.66	0.74
**Mn**	0.27	0.32	-	0.35	0.66	-	0.62
**Zn**	0.46	0.32	0.41	0.52	0.74	0.62	-

Table S2. Correlations between skin hydration and lubrication ($p < 0.05$).

Factors	H-Fa	H-Ne	H-Fh	H-No	H-Che	H-Chi	H-Ey	H-Total	L-Fh	L-No	L-Che	L-Chi	L-Total
**H-Fa**	-	0.52	0.16	-	0.26	0.28	-	0.46	-	-	-	-	-
**H-Ne**	0.52	-	0.23	-0.17	0.33	0.35	0.24	0.53	-	0.19	-	-	-
**H-Fh**	0.16	0.23	-	-	0.37	0.46	0.37	0.64	-	-	-	-	-
**H-No**	-	-0.17	-	-	-	-	-	0.34	-	-	-	-	-
**H-Che**	0.26	0.33	0.37	-	-	0.49	0.36	0.64	-0.26	-	-	-	-
**H-Chi**	0.28	0.35	0.46	-	0.49	-	0.37	0.74	-0.17	-	-0.15	-	-0.16
**H-Ey**	-	0.24	0.37	-	0.36	0.37	-	0.59	-0.30	-	-	-0.19	-0.22
**H-Total**	0.46	0.53	0.64	0.30	0.64	0.74	0.59	-	-0.19	-	-	-	-
**L-Fh**	-	-	-	-	-0.26	-0.17	-0.30	-0.19	-	0.32	0.46	0.50	0.73
**L-No**	-	0.19	-	-	-	-	-	-	0.32	-	0.52	0.44	0.75
**L-Che**	-	-	-	-	-	-0.15	-	-	0.46	0.52	-	0.48	0.79
**L-Chi**	-	-	-	-	-0.27	-	-0.19	-	0.50	0.44	0.48	-	0.76
**L-Total**	-	-	-	-	-0.24	-0.16	-0.22	-	0.73	0.75	0.79	0.76	-

H-Che – hydration of checks, H-Chi – hydration of chin, H-Ey– hydration of eyelids, H-Fa – hydration of forearm, H-Fh – hydration of forehead, H-Ne – hydration of neckline, H-No – hydration of nose, H-Total – hydration of all areas, L-Che – lubrication of cheeks, L-Chi – lubrication of chin, L-Fh – lubrication of forehead, L-No – lubrication of nose, L-Total – lubrication of all areas.
